# Correction to “Long Noncoding RNA OVAAL Enhances Nucleotide Synthesis Through Pyruvate Carboxylase to Promote 5‐Fluorouracil Resistance in Gastric Cancer”

**DOI:** 10.1111/cas.16444

**Published:** 2024-12-26

J.‐N. Tan, S.‐N. Zhou, W. Zhang, et al., “Long Noncoding RNA OVAAL Enhances Nucleotide Synthesis Through Pyruvate Carboxylase to Promote 5‐Fluorouracil Resistance in Gastric Cancer,” *Cancer Science* 113, no. 9 (2022): 3055–3070, https://doi.org/10.1111/cas.15453.

In the legend of Figure 5F of the published article, the authors made an inadvertent spelling mistake: “MGC823” needs to be corrected to “MGC803”.

The authors apologize for this error.

